# Applying user-centred techniques and expert feedback to refine an AI-based app for addressing mobile gaming addiction in adolescents

**DOI:** 10.1016/j.dialog.2025.100220

**Published:** 2025-04-28

**Authors:** Anna Khoziasheva

**Affiliations:** Ural Federal University named after the first President of Russia B.N.Yeltsin, Yekaterinburg, Russia

**Keywords:** Gaming addiction, mHealth, Mobile gaming disorder, Internet gaming disorder, Artificial intelligence, mHealth app, Online gaming

## Abstract

**Background:**

The prevalent use of smartphones has contributed to a rise in mobile gaming addiction, especially in young people. This study aimed to describe the design of an enhanced version of the AI-based mGaming Wellness mobile app, to support young individuals in developing healthy mobile gaming habits.

**Methods:**

The study utilised a 4-phased methodology, based on user-centred design principles, the Mobile App Rating Scale, a focus group and in-depth interviews with the app's target audience, and a think-aloud method.

**Results:**

The first round of refinement of mGaming Wellness, guided by input from an expert panel, focused on enhancing engagement and information quality. Feedback led to the identification of 5 key components for digital interventions, including mood and sleep trackers, a statistics dashboard, and educational modules tailored to young users' needs. Subsequent user research prompted the simplification of mood trackers and adjustments in educational content to align closely with adolescents' experiences. Usability testing of a high-fidelity prototype highlighted the app's ease of use and identified areas for further improvement, particularly in understanding how to reduce gaming time and effectively manage gaming-related notifications. The refined mGaming Wellness app can be a valuable resource for mental health professionals, educators, and youngsters seeking support with problematic mobile gaming or interested in building healthy digital habits.

**Discussion:**

The findings advocate for the user-centred techniques in developing digital health interventions, contributing valuable input for research in mental health app development targeted at adolescents. Future research will evaluate the app's effectiveness in reducing problematic gaming behaviour.

## 1 Introduction

Mobile devices have become an integral part of daily life, especially among young people. Smartphones and other mobile devices provide access to diverse activities from any place and at any time, shaping youngsters' social experiences and development [1]. The widespread use of smartphones has led to an increase in mobile gaming: approximately 3.4 billion people worldwide were playing video games in 2023 and it is expected that the number will to rise to 3.8 billion by 2026 [2]. Mobile gaming, a subset of video gaming, has become increasingly popular due to the convenience and accessibility of smartphones and tablets. Mobile gaming encompasses a broad range of game genres, including casual games like Angry Birds, competitive multiplayer games like Clash Royale, and immersive role-playing games such as Genshin Impact or World of Warcraft. Social games like Candy Crush Saga have also become cultural phenomena, attracting 93 million daily players and over 500 million installs on mobile devices, while generating substantial profits— Candy Crush Saga earned US$568 million in 2013 [3]. However, the widespread popularity of mobile gaming has also raised concerns about its potential for addiction. It was found that 7.3 % of Candy Crush Saga players in China were considered addicts.

With the growing popularity of games, problematic gaming among youth has become a major public health concern [4], with the worldwide prevalence of gaming disorder estimated to be between 1.96 % and 2.4 % [5].

Regional and age-specific variations in prevalence further illustrate the scale of the issue: approximately 8.5 % of U.S. youth aged 8–18 years meet criteria for gaming disorder, compared to 5.4 % of Dutch individuals aged 13–40, 1.2 % of German youth aged 13–18 years, and 5.9 % of South Korean adolescents aged 13–15 years [6].

In Russia, gaming has surged in popularity over recent years. As of 2023, 60 % of Russians aged 18 and older—approximately 88 million individuals—report playing video games either regularly or episodically, a figure that has more than tripled since 2018. [7] On average, Russian gamers spend 3–5 h per week playing video games, with 74 % of them preferring mobile devices such as smartphones or tablets. These trends highlight the increasing prevalence of gaming, particularly mobile gaming, and its growing role in shaping digital behaviour.

Gaming addiction is characterised by an individual's inability to stop playing games for long periods of time, responsibilities neglect and preferencing gaming over other activities. When such behaviour is repetitive, it has various negative effects on an individual's mental and physical health [8].

Many young individuals experience early symptoms of deteriorating mental health, which may progress to clinical disorder [9]. It's important to provide youth with tools for early screening and intervention to reduce this risk [10]. However, often young people do not seek help or find it difficult to get it, and this aspect of working with youth remains challenging [11].

Smartphone apps offer an opportunity and a platform for providing such support [12]. mHealth apps for adults already target depression, stress, panic, and anxiety [13] and apps for young people are also emerging [14,15]. The studies show that young people with mental health difficulties generally find mHealth apps acceptable, valuing the privacy and independence they offer [16].

According to the responsible digital research and innovation framework [17], mHealth apps for emerging adults should be co-designed with young people and professionals [18], educate on disorder triggers, symptoms, and their prevention and have user needs prioritised.

“mGaming Wellness” is an artificial intelligence (AI) based mobile application developed to address the risk of problematic gaming in young people by helping them monitor their gaming habits and assess their level of mobile gaming addiction. The app collects various data on a mobile device and then processes it using a neural network to evaluate the current risk of a mobile gaming addiction. Following Bandura's social cognitive theory (SCT) framework [19], mGaming Wellness supports youth by providing education and promoting healthy gaming habits. While the app demonstrated potential in assessing gaming addiction risk and received positive initial feedback regarding its functionality, early-stage user feedback revealed gaps limiting its ability to encourage behaviour change. In response to this need, the app was revised and improved based on expert feedback and user-centred (UCD) principles. UCD is a widely used method in developing software applications that improves and prioritises the needs and preferences of end users [20].

This study describes the process of designing an enhanced version of mGaming Wellness by applying the UCD approach. By exploring enhancements and their implications, this study contributes to the broader discourse on the effectiveness of digital interventions in managing behavioural issues associated with mobile gaming in youth.

## 2 Methods

### 2.1 Overall study design

This was a phenomenological study that took place in Russia and adopted a health belief model (HBM) approach [21]. The HBM states that there are 2 cognitive processes that determine a person's behaviour in response to a threat – how seriously one assesses the consequences of the threat and how effective and feasible the protective behaviour against the threat is [22]. The study's design was informed by HBM, ensuring that the app’ addresses users' perceived threats (susceptibility and severity) of mobile gaming addiction, while also highlighting the benefits and facilitating of healthier gaming habits adoption, as by so influencing users' behaviours and actions. Through this study, the aim was to analyse various stakeholders in order to understand how to best raise awareness about mobile gaming addiction, promote healthy digital behaviours, and educate on preventive health measures within a mobile app. The purpose of this study to describe the design of an enhanced version of the free AI-based mGaming Wellness mobile app, to support young individuals in developing healthy mobile gaming habits. To this end, a mixed-methods study involving expert evaluations, end-user feedback, and usability assessments was conducted. The research was organized into 4 distinct phases, based on the UCD framework (ISO 9241).

In the next sections, each phase of the research is examined with details related its design, participants, data collection, and data analysis.

### 2.2 Ethical considerations

At every phase of the research, the study's purpose was clearly communicated, and participants were guaranteed anonymity. Each participant provided written informed consent to participate in the study. For respondents under 18 years old, written consent was obtained from parents or guardians. Despite data protection measures, an ethical approval was acquired from the Ural Federal University's Institutional Review Board (IRB) prior to each point of data collection.

### 2.3 Phase 1: prototype development

Firstly, 31 field experts were recruited via a range of channels, including social networks, recommendations from universities and health professional organisations. The expert panel reviewed the existing version of mGaming Wellness using the Mobile App Rating Scale (MARS) [23]. The MARS was converted to a Google Forms questionnaire and distributed to participants via email. This research stage was held from September 23, 2024 to September 30, 2024 with the consent of the participants and prior approval from Ural Federal University's IRB.

The MARS is a recognised and used in mHealth tool that measures app quality across 4 subscales: engagement, functionality, aesthetics, and information quality. Each question is rated on a 5-point Likert scale (1 – lowest quality score: 5 – highest quality score).

Quantitative data from the MARS were analysed using SPSS v.29.0. Descriptive statistics (mean, standard deviation, median, interquartile range, and relative interquartile range) were calculated for each question to assess the distribution of expert ratings.

Secondly, a literature overview, using database searches (ScienceDirect, Scopus, PubMed, Web of Science, and Google Scholar) and manual searches, on computer and mobile gaming assessment and related intervention strategies was conducted. The search terms included combinations of keywords such as “mobile gaming addiction”, “internet gaming disorder”, “gaming disorder”, “gaming addiction interventions”, “mHealth interventions”, “digital health tools”, “intervention strategies”, “prevention approaches”, “adolescent”, and “youth”. As a result, app screens requiring updates were identified, as well as new features to implement.

Finally, based on both inputs, a low-fidelity prototype was created and presented the app's improved navigation sequences, and newly identified features.

### 2.4 Phase 2: user-centred research

A total of 9 participants were recruited via experts participating in phase 1 and health professional organisations. These participants were youngsters who were self-reported mobile gamers (engaged in playing mobile games for at least 3 h per week) and expressed interest in contributing to the development of tools for addressing mobile gaming addiction. The research purpose was carefully explained and each respondent provided written informed consent to participate. Respondents under 18 years old provided a written consent from their parents or guardians. Data collection was conducted with a prior approval from Ural Federal University's IRB. This phase took place from 5 December 2024 to 16 December 2024.

A focus group (*n* = 3) and structured interviews (*n* = 6) were conducted to explore perceived barriers and facilitators to the use of mHealth tools and to obtain feedback on the prototype. While the sample size was small, it was sufficient for the exploratory nature of this study, which aimed to gain initial insights into user experiences and preferences. Future studies will employ larger samples to validate findings and assess broader efficacy of the app enhancements.

Feedback was sought on the app screens, layout, and navigation; features usefulness; usefulness of educational materials; clarity of data visualizations; and additional features and content.

All interviews were audio-recorded and transcribed verbatim. A thematic analysis in Atlas.ti was performed to create interview themes [24].

### 2.5 Phase 3: usability testing

The usability testing with 7 adolescents recruited via health worker organisations was carried from December 22, 2024 to December 30, 2024. The research objective was thoroughly explained and each respondent provided written informed consent (for those under 18 years old, their parents/guardians). An approval was sought from Ural Federal University's IRB for this research phase.

For this phase, a high-fidelity interactive prototype for the entire app was created using experts' and users' feedback. The prototype was user-tested with adolescents via the Think-Aloud (TA) method [25]. Individual 1-on-1 usability testing sessions were conducted online. Usability testing consisted of 2 steps [26]. The respondents were asked to share verbally their thoughts as they navigated through the prototype and completed tasks. Participants were also asked to rate the difficulty of completing each task on a 5-point scale (1 – difficult, 5 – very easy). Finally, they were asked to share their opinion on how the app could be improved [27].

Content analysis in Atlas.ti was used to analyse and summarise the notes and verbalisations from the TA [28], focusing on predefined categories (usability issues, user experience, suggestions for improvements).

### 2.6 Phase 4: the revised app version

The final phase consisted of a descriptive compilation of key findings from the previous phases, integrating expert evaluations, user feedback, and usability test results. The notes and observations from the usability testing were scanned for indicators of usability issues. As a result, a list of recommended modifications was compiled.

No additional statistical or thematic analyses were conducted, as the primary goal was to synthesise recommendations for the app's revision.

The results will be used by the app developers to address the key usability barriers and users' preferences and feedback and develop a refined version of the mGaming Wellness app for testing in a pilot clinical trial.

**Fig. 1:**
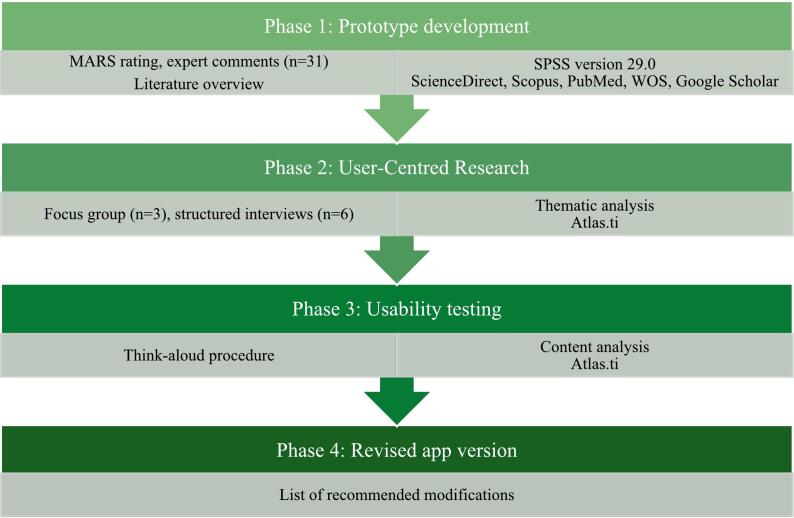
Methodological flow.

The full methodological flow is presented on Fig. 1.

## 3 Results

The intervention components of the app were developed through an iterative process involving expert feedback and user-centred design principles. Initial features were obtained from expert panel and relevant literature. These features were then refined using feedback from focus groups, interviews, and usability testing with emerging adults.

### 3.1 Initial prototyping

The first input into the initial prototype for the app refining was from the expert panel of 31 participants. The socio-demographic statistics are presented in Table 1. This group provided their written feedback on the app in the Google Forms survey while rating the app on the MARS in the open-ended section.

**Table 1:** Expert panel description.

Characteristic	Respondents (*n* = 31)
Gender	
Male	11 (35.48 %)
Female	20 (64.52 %)

Age	
31–40	10 (32.26 %)
41–50	8 (25.81 %)
51–60	12 (38.71 %)
61–70	1 (3.23 %)

Respondent profile	
Clinician or psychologist	19 (61.29 %)
Researcher	3 (9.68 %)
Technology-related position	4 (12.90 %)
Consultant or similar position	5 (16.13 %)

Self-reported knowledge of health apps	
1 (low)	1 (3.23 %)
2	3 (9.68 %)
3	5 (16.13 %)
4	14 (45.16 %)
5 (expert)	8 (25.81 %)

**Fig. 2:**
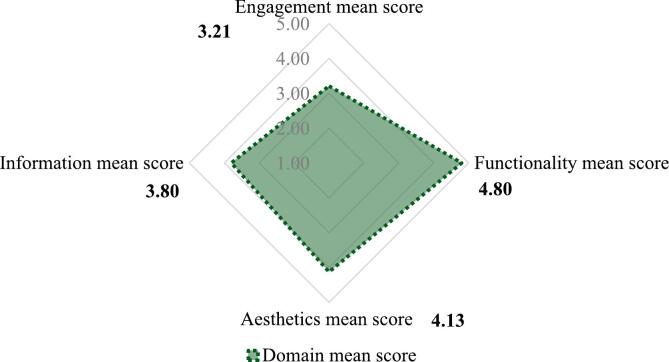
MARS items average scores.

According to the experts' MARS rating (see Fig. 2), the app demonstrates strong points in functionality (SD = 4.80), and aesthetics (SD = 4.13) with positive comments in areas such as ease of use, visual appeal, and promoting behaviour change. However, the app received a moderate score for engagement (SD = 3.21). After analysing remarks left in the open-ended sections, it was concluded that the app's ability to keep user interest needs to be enhanced. Finally, the experts left comments that enriching the quality of information (SD = 3.80) provided is crucial for the app.

In the next step, through a process of reviewing the relevant literature and theory-based behavioural strategies review and results from the expert panel, 5 key intervention components for the app were identified. The key components are described below.

**Component 1: daily mood and sleep trackers.** Addicted gamers tend to have a greater risk of feeling irritable or in a bad mood, feeling nervous and exhausted [29]. Unpleasant emotions are also observed during a withdrawal. Addiction to game playing is linked to negative effects on sleep quality and timing, including delayed sleep onset [30]. Sleep patterns and mood changes can provide invaluable insights into the early warning signs of mobile gaming addiction.

**Component 2: dashboard with statistics.** Visualising gaming data and health metrics can promote self-awareness and healthier behavioural patterns [31] so that young individuals can achieve a clearer understanding of the impacts of gaming on their physical and emotional well-being, encouraging them to evaluate their gaming habits critically.

**Component 3: notification muting.** Notifications may serve as triggers for gaming cravings, undermining self-control and the ability to focus on important tasks or activities [32]. Muting gaming and other notifications is a crucial strategy in reducing these triggers. Disabling notifications may provide periods of “digital detox” – an opportunity to get involved in non-digital activities. Furthermore, this strategy supports the adoption of healthier digital habits, helping to develop a more balanced relationship with technology.

**Component 4: educational module.** It was suggested by the expert panel to enhance the app's knowledge base and educational resources. Adolescents, educated with knowledge about the signs, symptoms, and consequences of gaming addiction, are better equipped to self-reflect and recognise potentially harmful behaviours and gaming patterns [33]. Educational resources can provide practical strategies for managing screen time and promoting healthier engagement with technology.

**Component 5: tracker for days without playing games.** The feature that tracks consecutive days a user spends without gaming can serve as a tangible form of reinforcement, showing the progress that adolescents are making as they attempt to reduce their time spent gaming [34]. By visualising the amount of time not spent gaming, adolescents can set more precise goals, celebrate milestones of abstinence or reduced gaming, and build motivation to continue their efforts. Encouraging a mindful and moderated approach to gaming can assist adolescents in developing healthier digital habits, supporting their overall well-being and preventing the escalation of problematic gaming.

### 3.2 User research

Qualitative findings from the focus group and interviews provided insight into the app content and features, as well as design elements that needed to be added or modified. Briefly, all participants agreed that the proposed mobile app and features would be useful and relevant to adolescents interested in gaming in general and in mobile games in particular, yet some changes were suggested.

**Fig. 3:**
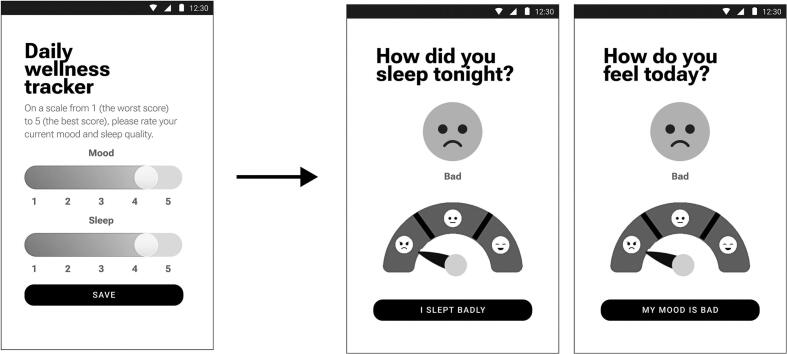
UI changes for the trackers.

The daily mood tracker was designed as a feature allowing users to record their emotional state at various times during the day, using a slider scale with emoticon representations. Feedback from users indicated that the mood tracker was helpful for self-awareness of emotional fluctuations. Users appreciated the simplicity of the input method, but faced difficulties with the scale itself (“…trying to differentiate between the nuances of a 4 and a 5… is quite challenging. Similarly, deciding if a 1 or a 2…”; “I find myself overthinking the distinctions between these points, and … I lose the willingness to rate my mood and sleep quality at all.”). Based on this finding, the mood and sleep trackers were simplified by introducing a 3-point scale instead of a 5-point (See Fig. 3).

**Fig. 4:**
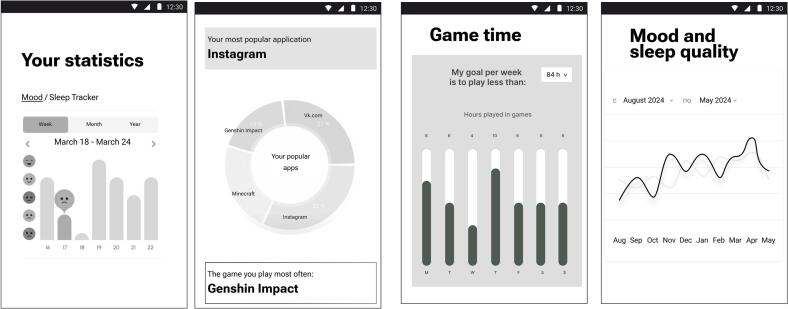
Several graphs and charts from the analytical dashboard.

The dashboard provided detailed statistics related to gaming and non-gaming activities, presenting data in various formats such as graphs, timelines, and numerical indicators (see Fig. 4.). Test participants found the dashboard informative and motivating. The participants noted that it would help them understand their gaming habits better and encourage them to set limits (“If the dashboard … allows me to set gaming goals or challenges, I would check it more often. Like in games, I enjoy checking progress towards these goals … it would motivate me to return regularly”). The visual representation of data was praised for its user-friendly interface (“… checking out my gaming habits feels more like fun than analysis”). While users described the dashboard statistics as “fun” and noted they might check it multiple times a day, this feature was designed to encourage self-awareness rather than excessive app usage. By gamifying self-monitoring behaviours, the dashboard aims to promote healthier gaming habits without inadvertently increasing screen time. The users noted this can be the most visited screen of the app, with users checking their statistics multiple times a day.

The knowledge base included resources like articles, useful websites, and hotlines for youth. The content was well-received, with users noting that it provided valuable insights (“… recognising gaming addiction signs was really interesting. I never realised some of my habits might be problematic until I saw them explained like that”). In addition, the educational module was revised to include materials from youngsters themselves talking about gaming addiction.

**Fig. 5:**
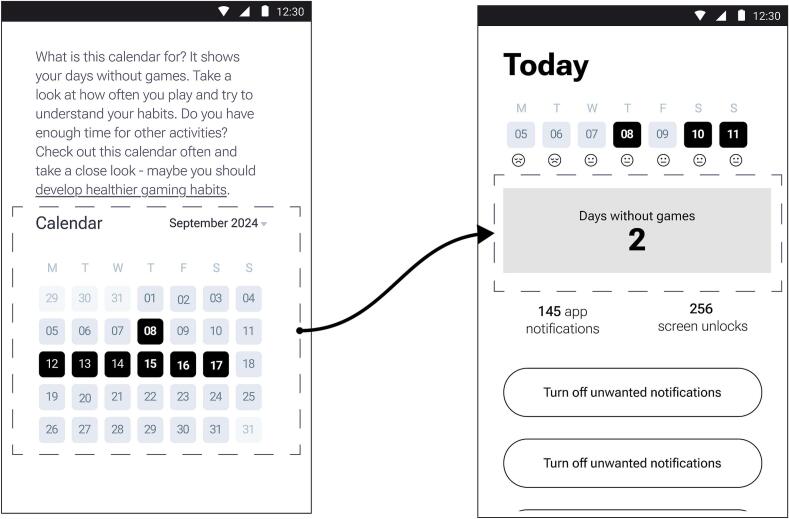
Improved version of the consecutive days without gaming tracker.

Based on target users' suggestions, the no gaming days tracker (see Fig. 5) was changed to show the total number of consecutive days without gaming, rather than the results of the last month (“… the calendar's bulky design makes it less appealing…”; “ I've accustomed to seeing summary counters, like I have seen in my other favourite apps”). Finally, based on findings from the qualitative study, some app design elements were updated.

### 3.3 Usability testing

**Fig. 6:**
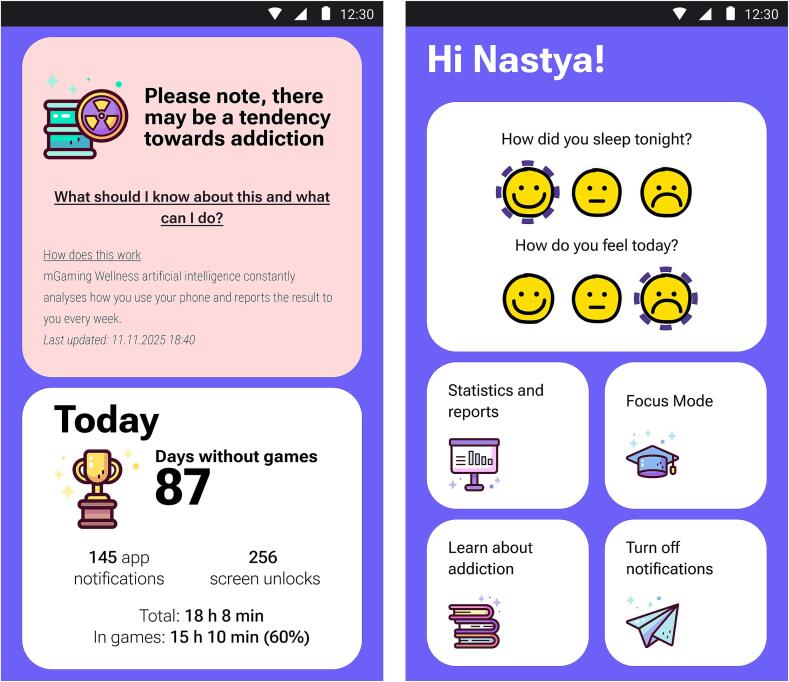
One of the interactive prototype screens.

A high-fidelity clickable prototype of the app (see Fig. 6) was built and incorporated aspects highly regarded by experts and users. In total, 7 users participated in the usability testing. The participants' mean age in the usability testing was 18.1 (SD 1.3) years. All participants were interested in mobile games and engaged in playing on mobile devices for at least 3 h per week).

Results from the TA are described in Table 2. The mean score across the 10 tasks was 4.13 (SD 1.09). The task completion rates ranged from 71.43 % (*n* = 5) to 100.00 % (*n* = 7) across 10 tasks, with the lowest completion rate for the task about understanding how to reduce gaming time.

**Table 2:** Usability testing of key features of the app among target users.

Successful completion, n (%)	Difficult, n (%)	Difficult, n (%)	Normal, n (%)	Easy, n (%)	Very easy, n (%)	Score, mean (SD)
**Task 1.** Register						
7 (100.0)	0 (0.0)	0 (0.0)	1 (14.29)	5 (71.43)	1 (14.29)	4.00 (0.53)
**Task 2.** Rate today's mood						
7 (100.0)	0 (0.0)	0 (0.0)	0 (0.0)	1 (14.29)	6 (85.71)	3.86 (0.35)
**Task 3.** Rate today's sleep						
7 (100.0)	0 (0.0)	0 (0.0)	0 (0.0)	1 (14.29)	6 (85.71)	3.86 (0.35)
**Task 4.** Understand the current risk towards addiction						
6 (85.71)	0 (0.0)	1 (14.29)	0 (0.0)	0 (0.0)	6 (85.71)	3.71 (0.70)
**Task 5.** Understand how to reduce gaming time						
5 (71.43)	1 (14.29)	1 (14.29)	5 (71.43)	0 (0.0)	0 (0.0)	2.57 (0.73)
**Task 6.** Understand where to learn more about gaming addiction						
7 (100.0)	0 (0.0)	0 (0.0)	0 (0.0)	0 (0.0)	7 (100.00)	5.00 (0.00)
**Task 7.** Find out if gaming time is reduced on increased recently						
6 (85.71)	0 (0.0)	1 (14.29)	1 (14.29)	5 (71.43)	0 (0.0)	3.57 (0.73)
**Task 8.** Find out what applications are most frequently used						
7 (100.0)	0 (0.0)	0 (0.0)	0 (0.0)	0 (0.0)	7 (100.00)	5.00 (0.00)
**Task 9.** Understand how to turn off gaming notification						
7 (100.0)	0 (0.0)	1 (14.29)	4 (57.14)	2 (28.57)	0 (0.0)	3.14 (0.64)
**Task 10.** Find out your connection between gaming patterns and sleep quality						
6 (85.71)	1 (14.29)	1 (14.29)	0 (0.0)	2 (28.57)	3 (42.86)	3.71 (1.48)

### 3.4 Suggested modifications to the app after usability testing

Findings from the expert panel feedback, user interviews, and usability testing were reviewed to identify recurring themes of feedback concerning mGaming Wellness' content, usability, navigation, and features. As shown in Table 3, these themes serve as valuable insights that will be used in the app development to address the key usability barriers and participants' feedback and develop the refined app version for running it in a pilot clinical trial.

**Table 3:** List of recommendations from Phases 1–3.

Area of improvement	Phase 1	Phase 2	Phase 3
Mood Tracker	Simplify scale to improve engagement	Adopt 3-point scale for ease of use (previously 5-point)	–
Sleep Tracker	Align with mood tracker simplicity	Adopt 3-point scale, similar to mood tracker	–
Statistics Dashboard	Enhance with more detailed analytics and visualizations	Include goal-setting features, user-friendly interface	Show dashboard as a key feature
Notification Management	Introduce notification muting to reduce gaming cues	Retain notification muting, emphasise digital detox	–
Educational Module	Expand resources, include practical strategies and user testimonials	Include materials from youngsters, clarify misconceptions	–
Tracker for Days Without Gaming	Show total consecutive days without gaming	Retain, with summary counter display	–
Reminders	–	–	Add reminders for each hour played during the day Add reminders to take a break during gaming sessions Add reminders to rate sleep and mood Get daily motivational messages Add “Go to bed” reminders if playing at a late time
Gaming Time Management	–	–	Set limits on daily or weekly gaming time Show trends if gaming behaviour is improving or deteriorating
Assessment Tools	–	–	Add more tests to assess gaming addiction symptoms
Digital Detox	–	–	Set digital detox days and periods with no gaming, encouraging engagement in other activities
Educational resources	–	–	Add an educational module on how family members or friends can provide support if facing gaming addiction symptoms Clarify common misconceptions about gaming addiction Add a video section with testimonies about gaming addiction Add articles on developing healthy habits and reducing gaming time Add practical advice on managing gaming behaviour

The decision to retain or disregard the requested modifications will be based on their potential influence on behavioural and clinical outcomes, feasibility, the implementation time frame, and their impact on the app's scalability in the future.

## 4 Discussions

Digital health tools are a reliable instrument for identifying problematic behavioural patterns and facilitating interventions [35]. The use of such tools is aligned with young people's digital environments.

In literature, there is growing advocacy for utilising mHealth tools as proactive measures in preventing and mitigating negative socioemotional outcomes [36,37]. Several studies [[38], [39], [40]] have praised digital interventions for their accessibility and potential to deliver personalised therapeutic strategies effectively, especially among youth struggling with gaming disorders.

In this paper, the design for the future enhanced version of mGaming Wellness, an AI mHealth app, based on the SCT framework, is described. mGaming Wellness evaluates the level of mobile gaming addiction and promotes healthy gaming habits in emerging adults. Enhancing mGaming Wellness was supported by a user-centred design approach, including field experts' and end-users' feedback to better understand and meet the target audience's needs. Studies show that the user-centred design approach is significant when developing mHealth tools, ensuring they are adjusted to the target audience's needs and preferences, and leading to more effective and engaging health interventions [41,42].

Through the integration of expanded functionalities and content, the mGaming Wellness app was noticeably improved in both user engagement and educational value.

First, mood and sleep trackers as a form of daily journaling that helps users in self-monitoring and critical for behaviour change [43], were designed. By understanding emotional states triggered by play, users can better manage their habits. This functionality aligns with studies [44,45] showing that young people addicted to gaming had a greater risk of feeling low, irritable, tired, and being in a bad mood.

Second, the dashboard with gaming and non-gaming statistics was a significant improvement in user engagement. This feature is supported by studies suggesting that providing users with feedback about their behaviour can promote self-regulation and mindfulness [46,47]. Users' positive feedback on the dashboard indicates its value in making invisible habits visible, empowering users to make informed decisions about their gaming behaviour.

Third, the muting notification setting proved to be an important feature. Empirical evidence suggests that reducing exposure to gaming cues, such as notifications, can decrease screen time, mitigate the risk of addiction, and assist users in achieving their goals towards healthier digital habits [48].

Finally, the knowledge base provided users with information about addiction triggers, coping strategies, and the psychological impacts of excessive gaming. Such a knowledge base increases digital competence and self-awareness. This, in turn, can facilitate self-reflection and complement other intervention strategies. Moreover, the knowledge base can help not only those already at risk but also informing their peers about how to support at-risk individuals [49,50].

Feedback from experts has helped to identify initial gaps and improve educational and interactive components. User feedback for the newly added features and improvements provided a broader insight into how well these align with real user needs and preferences, resulting in increased user satisfaction. Such a combined feedback approach contributed to the iterative design process, ensuring that improvements were relevant and reliable.

The mGaming Wellness app can be a valuable resource for a variety of stakeholders. Mental health professionals can apply the app in their practice, offering it as an additional tool for managing gaming habits. Educators can use it to nurture digital responsibility in students. Young individuals looking to reduce problematic gaming behaviour or develop healthy digital habits may find the app a useful and educational resource.

## 5 Future work and limitations

Although improvements to mGaming Wellness have shown promising initial results, there are several areas of future work and considerations regarding limitations that need to be highlighted when understanding the results of the study.

The current review and testing were limited to a group of users from Russia. This group may not represent the broader population of mobile game users or reflect the full range of severity of gaming addiction. In addition, a structured system was not used to modify and optimise the application. Yet prespecified criteria, including feasibility, scalability, and affordability, are aligned with existing frameworks designed to optimise and evaluate an intervention prior to its implementation [51,52].

In the future, it is planned to improve the app by developing the features and modifications found during the research process. After that, a longitudinal study of the app's effectiveness in assessing and preventing mobile game addiction over time needs to be conducted. This will help to understand the impact and durability of behavioural changes induced by the use of the app. In addition, continuous improvement of the app will be based on user feedback, which will allow adjusting its functionality and content according to user needs.

## 6 Conclusion

This study highlights the role of including both expert feedback and user-centred design principles in the development of mHealth mobile apps. Through feedback and targeted improvements, the mGaming Wellness app has expanded its functionality and user interface, becoming a more effective tool for assessing mobile gaming addiction in young people.

Enhanced features, namely daily mood and sleep trackers, a dashboard with analytical statistics, customisable notification settings and an enriched knowledge base, have been developed based on expert opinions and user preferences. The positive changes observed in user feedback indicate the acceptability, improved usability, and user satisfaction with these enhancements. While this study focused on the usability and design process of the mGaming Wellness app, the next step is to evaluate its effectiveness in achieving its intended outcomes. Future research will involve a longitudinal study to assess the app's ability to reduce problematic gaming behaviours, promote healthy digital habits, and support mental well-being in its target audience.

This study contributes to the current literature on evaluating and addressing mobile gaming addiction in children and adolescents. By documenting the enhancement process with expert and end-user feedback, this work provides a valuable contribution to design strategies that can optimise mobile health applications for better user acceptance and improved effectiveness.

## Research involving human participants—rights

The study involved human participants who voluntarily chose to participate in the research. All research participants are guaranteed privacy and anonymity.

## CRediT authorship contribution statement

**Anna Khoziasheva:** Writing – review & editing, Writing – original draft, Visualization, Validation, Project administration, Methodology, Formal analysis, Conceptualization.

## Informed consent

All respondents provided written informed consent before their participation. For respondents less than 18 years old, written consent was obtained from parents or guardians. Participation in the study was voluntary. The data was collected, saved and analysed anonymously.

## Ethical approval

All procedures followed were in accordance with the ethical standards and principles of conducting research at Ural Federal University in Ekaterinburg, Russia. The study was granted ethical approval from the relevant Research Committee.

## Funding

This research did not receive any specific grant from funding agencies in the public, commercial, or not-for-profit sectors.

## Declaration of competing interest

The author has no relevant financial or non-financial interests to disclose.

## Data Availability

All data and materials claims and comply with field standards. The data generated during and/or analysed during the current study are available from the corresponding author upon reasonable request.

## References

[1] Livingstone S., Smith P.K. (2014). Annual research review: harms experienced by child users of online and mobile technologies: the nature, prevalence and management of sexual and aggressive risks in the digital age. J of Child Psychology and Psychiatry.

[2] Global Games Market Report 2023 | May 2024 Update. Newzoo. Accessed April 6, 2025. https://newzoo.com/resources/trend-reports/newzoo-global-games-market-report-2023-free-version.

[3] Chen C., Leung L. (2015). Are you addicted to Candy Crush Saga? An exploratory study linking psychological factors to mobile social game addiction. Telematics and Informatics.

[4] Rosendo-Rios V., Trott S., Shukla P. (2022). Systematic literature review online gaming addiction among children and young adults: A framework and research agenda. Addictive Behav.

[5] Stevens M.W., Dorstyn D., Delfabbro P.H., King D.L. (2020). Global prevalence of gaming disorder: a systematic review and meta-analysis. Australian & New Zealand Journal of Psychiatry.

[6] Kim H.S., Son G., Roh E.B. (2021). Prevalence of gaming disorder: a meta-analysis. Addict Behav.

[7] NAFI Research Center. GAMING IN RUSSIA - 2022. Social and Economic Effects - NAFI. NAFI Research Center. Accessed April 6, 2025. https://nafi.ru/projects/it-i-telekom/geyming-v-rossii-2022-sotsialnye-i-ekonomicheskie-effekty.

[8] Zhang M.X., Lam L.W., Wu A.M.S. (2022). Recovery experiences protect emotionally exhausted white-collar workers from gaming addiction. Intern J of Environ Res and Public Health.

[9] Kim-Cohen J., Caspi A., Moffitt T.E., Harrington H., Milne B.J., Poulton R. (2003). Prior juvenile diagnoses in adults with mental disorder. Archives of Gen Psychiatry.

[10] Hugh-Jones S., Pert K., Kendal S., Eltringham S., Skelton C., Yaziji N. (2022). Adolescents accept digital mental health support in schools: a co-design and feasibility study of a school-based app for UK adolescents. Mental Health & Prevention.

[11] Holmes E.A., Ghaderi A., Harmer C.J., Ramchandani P.G., Cuijpers P., Morrison A.P. (2018). The Lancet Psychiatry Commission on psychological treatments research in tomorrow’s science. Lancet Psychiatry.

[12] Van Ameringen M., Turna J., Khalesi Z., Pullia K., Patterson B. (2017). There is an app for that! The current state of mobile applications (apps) for DSM-5 obsessive-compulsive disorder, posttraumatic stress disorder, anxiety and mood disorders. Depress Anxiety.

[13] Okoro N.Y.O., Ayo-Farai N.O., Maduka N.C.P., Okongwu N.C.C., Sodamade N.O.T. (2024). The role of technology in enhancing mental health advocacy: a systematic review. International J of Appl Res in Soc Sci.

[14] Leech T., Dorstyn D., Taylor A., Li W. (2021). Mental health apps for adolescents and young adults: a systematic review of randomised controlled trials. Children and Youth Services Rev.

[15] Jones R.B., Thapar A., Stone Z., Thapar A., Jones I., Smith D. (2017). Psychoeducational interventions in adolescent depression: a systematic review. Patient Educ Couns.

[16] Grist R., Cliffe B., Denne M., Croker A., Stallard P. (2018). An online survey of young adolescent girls’ use of the Internet and smartphone apps for mental health support. BJPsych Open.

[17] Jirotka M., Grimpe B., Stahl B., Eden G., Hartswood M. (2017). Responsible research and innovation in the digital age. Commun of the ACM.

[18] Jones R.B., Thapar A., Rice F., Mars B., Agha S.S., Smith D. (2020). A digital intervention for adolescent depression (MoODHWB): mixed methods feasibility evaluation. JMIR Mental Health.

[19] Bandura A. (1991). Social cognitive theory of self-regulation. Organ Behav and Hum Decis Process.

[20] Farao J., Malila B., Conrad N., Mutsvangwa T., Rangaka M.X., Douglas T.S. (2020). A user-centred design framework for mHealth. PloS One.

[21] Conner M., Norman P. (2005). Predicting health behaviour.

[22] Ahadzadeh A.S., Pahlevan S.S., Ong F.S., Khong K.W. (2015). Integrating health belief model and technology acceptance model: an investigation of health-related internet use. J Med Internet Res.

[23] Stoyanov S.R., Hides L., Kavanagh D.J., Zelenko O., Tjondronegoro D., Mani M. (2015). Mobile App Rating Scale: a new tool for assessing the quality of health mobile apps. JMIR Mhealth Uhealth.

[24] Kiger M.E., Varpio L. (2020). Thematic analysis of qualitative data: AMEE Guide 131. Med Teach.

[25] Rubin J., Chisnell D. (2011).

[26] Lewis J.R. (2018). The system usability scale: past, present, and future. Intern J of Hum-Comput Interact.

[27] Zhou L., Bao J., Setiawan I.M.A., Saptono A., Parmanto B. (2019). The MHealth App Usability Questionnaire (MAUQ): development and validation study. JMIR Mhealth Uhealth.

[28] Erlingsson C., Brysiewicz P. (2017). A hands-on guide to doing content analysis. African J of Emerg Med.

[29] King D.L., Delfabbro P.H., Griffiths M.D., Gradisar M. (2012). Cognitive-behavioral approaches to outpatient treatment of internet addiction in children and adolescents. J Clin Psychol.

[30] Owens J.A., Weiss M.R. (2017). Insufficient sleep in adolescents: causes and consequences. Minerva Pediatrics.

[31] King D.L., Delfabbro P.H., Billieux J., Potenza M.N. (2020). Problematic online gaming and the COVID-19 pandemic. J of Beh Addctn.

[32] Twenge J.M., Campbell W.K. (2018). Associations between screen time and lower psychological well-being among children and adolescents: evidence from a population-based study. Prev Med Rep.

[33] Lui J.C., Sagar-Ouriaghli I., Brown J.S.L. (2022). Barriers and facilitators to help-seeking for common mental disorders among university students: a systematic review. J of Am Coll Health.

[34] Billieux J., King D.L., Higuchi S., Achab S., Bowden-Jones H., Hao W. (2017). Functional impairment matters in the screening and diagnosis of gaming disorder. J of Beh Addctn.

[35] Maqbool B., Herold S. (2023). Potential effectiveness and efficiency issues in usability evaluation within digital health: a systematic literature review. J of Syst and Softw.

[36] Opie J.E., Vuong A., Welsh E.T., Gray R., Pearce N., Marchionda S. (2024). Outcomes of best-practice guided digital mental health interventions for youth and young adults with emerging symptoms: part I. A systematic review of socioemotional outcomes and recommendations. Clin Child and Family Psychology Rev.

[37] King D.L., Chamberlain S.R., Carragher N., Billieux J., Stein D., Mueller K. (2020). Screening and assessment tools for gaming disorder: a comprehensive systematic review. Clin Psychology Rev.

[38] Lindenberg K., Kindt S., Szász-Janocha C. (2022). Effectiveness of cognitive behavioral therapy–based intervention in preventing gaming disorder and unspecified internet use disorder in adolescents. JAMA Netw Open.

[39] He M., Ren Y., Liu Y., Tong K.K. (2024). The prospective effect of protective gaming beliefs and behaviors on problematic gaming, mental health, and well-being of video game players. Intern J of Hum-Comput Interact.

[40] Boumparis N., Haug S., Abend S., Billieux J., Riper H., Schaub M.P. (2022). Internet-based interventions for behavioral addictions: a systematic review. J of Beh Addctn.

[41] Elkes J., Cro S., Batchelor R., O’Connor S., Yu L.-M., Bell L. (2024). User engagement in clinical trials of digital mental health interventions: a systematic review. BMC Med Res Methodol.

[42] Lopes A., Valentim N., Moraes B., Zilse R., Conte T. (2018). Applying user-centered techniques to analyze and design a mobile application. J of Softw Eng Res and Dev.

[43] Lee H., Seo M.J., Choi T.Y. (2016). The effect of home-based daily journal writing in Korean adolescents with smartphone addiction. J of Korean Med Sci.

[44] Gentile D.A., Choo H., Liau A., Sim T., Li D., Fung D. (2011). Pathological video game use among youths: A two-year longitudinal study. Pediatrics.

[45] Mentzoni R.A., Brunborg G.S., Molde H., Myrseth H., Skouverøe K.J.M., Hetland J. (2011). Problematic video game use: estimated prevalence and associations with mental and physical health. Cyberpsychology Behav and Soc Netw.

[46] Roffarello A.M., De Russis L. (2022). Achieving digital wellbeing through digital self-control tools: a systematic review and meta-analysis. ACM Trans on Comput-Hum Interact.

[47] Ke C.L., Shih-Tsung C. (2019). Applied mindfulness smartphone app helps mitigating smartphone addiction. Eur Neuropsychopharmacol.

[48] Kim J., Cho C., Lee U. (2017). Technology supported behavior restriction for mitigating self-interruptions in multi-device environments. Proc of the ACM on Interact Mob Wearable and Ubiquitous Technol.

[49] Tso W.W.Y., Reichert F., Law N., Fu K.W., De La Torre J., Rao N. (2022). Digital competence as a protective factor against gaming addiction in children and adolescents: a cross-sectional study in Hong Kong. The Lancet Reg Health - West Pac.

[50] Chau C.-L., Tsui Y.Y.-Y., Cheng C. (2019). Gamification for Internet gaming disorder prevention: evaluation of a wise IT-USE (WIT) program for Hong Kong primary students. Front in Psychology.

[51] Collins L.M., Murphy S.A., Strecher V. (2007). The multiphase optimization strategy (MOST) and the sequential multiple assignment randomized trial (SMART). Am J of Prev Med.

[52] Michie S., Van Stralen M.M., West R. (2011). The behaviour change wheel: a new method for characterising and designing behaviour change interventions. Implement Sci.

